# First-in-human, phase I single-ascending-dose study of the safety, pharmacokinetics, and relative bioavailability of selatinib, a dual EGFR-ErbB2 inhibitor in healthy subjects

**DOI:** 10.1007/s10637-020-00959-6

**Published:** 2020-06-13

**Authors:** Meng-na Wang, Yun Kuang, Li-ying Gong, Ye Hua, Qi Pei, Cheng-xian Guo, Yu Cao, Jie Huang, Guo-ping Yang

**Affiliations:** 1 Center for Clinical Pharmacology, The Third Xiangya Hospital, Central South University, Changsha, Hunan 410013 People’s Republic of China; 2 Research Center of Drug Clinical Evaluation of Central South University, Changsha, Hunan 410013 People’s Republic of China; 3 Department of Cardiology, The Third Xiangya Hospital, Central South University, Changsha, Hunan 410013 People’s Republic of China; 4 Department of Pharmacy, The Third Xiangya Hospital, Central South University, Changsha, Hunan 410013 People’s Republic of China

**Keywords:** Tyrosine kinase inhibitor, Selatinib, First-in-human, Safety, Pharmacokinetics

## Abstract

We assessed the pharmacokinetics and safety of a single oral administration of selatinib to healthy Chinese subjects and evaluated the potential bioavailability advantage of selatinib relative to lapatinib. Healthy subjects aged 18–40 years were enrolled in this two-part study: Part 1, a single ascending dose (50–500 mg), randomized, double-blind, placebo-control study with 64 subjects; and Part 2, an open-label, positive control, randomized, three-treatment, three-period, three-sequence crossover design study, with 6 subjects administered a single 500-mg dose of selatinib tablets (A), selatinib suspension (B), or lapatinib tablets C) per cycle. In part 1, selatinib was well-tolerated up to the planned maximum dose of 500 mg; thus the maximum tolerated dose was not attained. Twenty-two adverse events were observed in 19 (36.5%) of the 52 subjects administered the test drug. The most common drug-related adverse event was diarrhea. The mean selatinib peak plasma concentration was 69.4–494 ng/mL, which was achieved in a median peak time of 3.5–4.5 h, with a mean elimination half-life between 13.8 and 15.8 h. In Part 2, A and B showed similar bioavailability. Plasma exposure to the active drug (selatinib plus the metabolite, lapatinib) after A intake was more than two-fold higher than that of the same dose of C. In the dose range of 50–500 mg, selatinib was safe and well-tolerated by healthy Chinese subjects, and it conformed with linear pharmacokinetics. Active exposure to selatinib was much greater than that to lapatinib, supporting its development as an adjuvant for anticancer treatment.

**Electronic supplementary material:**

The online version of this article (10.1007/s10637-020-00959-6) contains supplementary material, which is available to authorized users.

## Introduction

Selatinib ditosilate, an oral, reversible, dual inhibitor of epidermal growth factor receptor (EGFR) and ErbB2 tyrosine kinase receptors, has recently entered clinical development as a potential therapeutic agent for cancer. EGFR and ErbB2 are receptors tyrosine kinases of the ErbB family that consist of four closely related receptors: EGFR, ErbB2, ErbB3, and ErbB4 [1, 2]. EGFR and ErbB2 are known to contribute to normal and neoplastic growth processes in humans. Several tumors are known to exhibit increased EGFR activity, possibly because of increased synthesis, overexpression, or mutation of EGFR [3, 4]. EGFR transactivation in cancer cells can confer properties, such as growth, survival, and angiogenesis to cancer cells [5]. Previously,a study revealed that both EGFR expression and gene copy number variations are associated with poor prognosis of cancers [6]. The expression level of the downstream signal of the ErbB 2-mediated signaling pathway indicates that overexpression of the ErbB2 receptor leads to actin cytoskeleton remodeling, alterations in cell adhesion, and increased exercise capacity and invasiveness, leading to the transfer and escape of anti-tumor immunity [7]. Furthermore, overexpression of ErbB2 is associated with poor clinical outcome, poor prognosis, metastatic brain tumor and shorter survival [8–13].

Anticancer drugs targeting ErbB can be divided into two main categories: small-molecule tyrosine kinase inhibitors (TKIs) and monoclonal antibodies (mAb). Lapatinib is the most commonly used dual EGFR-ErbB2 TKIs approved both locally and internationally. In contrast to mAb inhibitors, lapatinib has less cardiotoxicity and better safety [14]. Several studies have demonstrated that compared to simple endocrine therapy or chemotherapy, combination therapy with lapatinib significantly prolongs the median time to progression, reduces the risk of disease progression, and improves the clinical benefit rate [15–17]. Furthermore, as a small molecule, lapatinib has been demonstratd to significantly inhibit metastatic encephaloma growth and significantly reduce the number of brain metastases [18, 19]. However, few patients respond to lapatinib, and they exhibit individual differences [20, 21]. No evidence has been found for drug-resistant cells within the nervous system isolated from lapatinib-treated brains, confirming that this minimal therapeutic effect is due to poor water solubility and low bioavailability [22, 23].

The test drug, selatinib [24] (Fig. S1), is a class 1.1 new drug. Compared to lapatinib, the water solubility of selatinib has been markedly improved to meet the solubility requirements of oral drugs.Though pharmacokinetic (PK) experiments [24] in animals, selatinib was found to have better absorption, higher bioavailability, and less individual variation relative to lapatinib. Additionally, a previous pharmacodynamic study [25] in vitro and in vivo confirmed that selatinib and lapatinib exhibited the same dual EGFR/ErbB2 inhibition specificity, with IC_50_ values of 13.0/22.5 and 16.3/37.4 nM, respectively. Furthermore, a preclinical toxicology study [24] revealed that it has lower liver toxicity and skin toxicity and is well-tolerated compared to lapatinib.

Therefore, this first-in-human study was conducted to determine the safety, tolerability, and PK profile of selatinib in healthy Chinese subjects.

## Materials and methods

### Statement of human rights

This study was reviewed and approved by the independent Ethics Committee of the Third Xiangya Hospital of Central South University (ethical approval numbers, 2012 L01883 and 2012 L01879). The study was also conducted according to the Declaration of Helsinki and Good Clinical Practice guidelines (GCP). All subjects gave written, informed consent before any study-related procedures were performed. The study was registered with the China Clinical Trials Registry(number ChiCTR1900027883).

### Subjects

Healthy Chinese subjects were screened for eligibility at ~1 week before administration of the test or reference drug. Screening included vital signs, medical history, 12-lead electrocardiogram (ECG) recording, physical examination, and laboratory test (hematology, urinalysis, and serology). Eligibility criteria included healthy Chinese adults, 18–40 years old (age difference in the same batch was no more than 10, with a body mass index (BMI) of 19–24 kg/m^2^ and a minimum weight of 50 and 45 kg for male and female subjects, respectively). Subjects did not have a history of cardiovascular, endocrine, metabolic, neurological, gastrointestinal, hepatic, pulmonary, infectious, immunological, or psychiatric disease. Subjects with a history of alcohol abuse, cigarette or drug dependence, and the use of concomitant treatments, defined as within 30 days of using any drug that inhibits/induces hepatic metabolizing enzymes or any investigational drug within four weeks of post-surgery, were excluded. Additionally, female subjects who were pregnant or planning on conceiving and those using oral contraception or menstruating were not suitable for this study.

### Study drug

Qilu Pharmaceutical Co., Ltd. (Shandong, China) supplied the selatinib ditosilate tablets (50, 100, and 250 mg/tablet; lot 2,012,080,202, 2,012,080,203, and 2,012,080,204,respectively). The selatinib ditosilate dry suspension (250 mg, lot 140,401) was employed as the test drug. Qilu Pharmaceutical Co., Ltd. supplied the selatinib ditosilate placebo (50, 100, and 250 mg/tablet; lot 2,012,083,002, 2,012,083,003, and 201,208,300,respectively) as the placebo-controlled drug. The lapatinib ditosylate tablets used as the positive reference drug (250 mg/tablet, lot R636591) were supplied by Glaxo Operations UK Limited (Brentford, UK).

### Study design

This study consisted of two parts.The randomization lists for Part 1 and Part 2 were created using a validated, automated system.

#### Part 1.

This was a single-center, randomized, double-blind, placebo-controlled, single ascending study involving healthy Chinese subjects. Subjects were randomly assigned to be adminitered either a single dose of selatinib or placebo. Investigators and subjects were blinded to the group allociations. Treatment identity was concealed by providing drug doses and placebo of identical appearance and in identical packageing. The starting dose was calculated according to the no observed adverse effect level (NOAEL) for animals.^[[25, 26^^]^ To derive the NOAEL of an adult with a body weighr of 60 kg, we converted the NOAEL of beagle dogs and Sprague-Dawley rats(i.e.,75 and 15 mg/kg, respectively) to human NOAEL doses of 498 and 726 mg, respectively. The starting dose should be 1/10 of the human NOAEL(i.e., starting dose was 50 or 72 mg). This dose was used as a threshold guide for dose escalations. To minimize risk while providing some inhibitory effects, 50 mg was recommended for use as the starting dose. Dose escalation was designed according to the modified-Fibonacci sequence. A total of 7 dose groups were included. Sixty-four subjects were administered 50 mg (*n* = 6), 100 mg (*n* = 12), 200 mg (n = 12), 250 mg (*n* = 10), 300 mg (n = 12), 350 mg (*n* = 8), or 500 mg (*n* = 4) selatinib tablets or placebo. Each group consisted of 50% males and females. In addition to the 500 mg group, 2 subjects in each group were administered a single dose of placebo, one for each gender. Only 2 subjects were initially enrolled in all dose groups. Follow-up subjects were enrolled on the premise that the drug was safely tolerated at the administration dose. When all subjects in the current dose group completed the trial and this dose was deemed as tolerable, the next dose group was initiated.

Subjects (*n* = 64) were randomized to be administered a single dose of oral placebo or selatinib tablets after overnight fasting for at least 10 h. Tablets were administered with 240 mL of tap water at ambient temperature. Consumption of additional water was not permitted within 1 h before and after drug administration. Light meals were served at 4 and 10 h after each dose, with identical standardized diets provided across all dosing periods.

If ≥3 of the 6 subjects in the 50 mg dose group had a “prescribed adverse event”, the trial was planned to be discontinued; and If ≥4 of the 8 subjects or ≥ 5 of the 10 subjects or ≥ 6 of the 12 subjects in the 100–350 mg dose group had a “prescribed adverse event”, the trial was planned to be discontinued. In the 500-mg dose group, if ≥3 of the 6 subjects had a “prescribed adverse event”, the trial was planned to be discontinued. Thereafter, the investigator and sponsor would jointly decide whether to terminate the study.

#### Part 2.

This was a randomized, positive control, open, single-center study with a three-cycle crossover design in healthy Chinese subjects. A total of 6 male subjects were randomized to one of the three treatment sequences (three-sequence crossover: ABC, BCA, or CAB [A, selatinib ditosilate tablet; B,selatinib ditosilate dry suspension; and C,lapatinib ditosilate tablet]). The wash-out period was 7 days.

The method employed for tablet administration was consistent with that in Part 1. The following method was employed to administer reference drug B: first, selatinib ditosilate (containing selatinib 500 mg) and 653 mg lactose T80 were accurately weighed. Thereafter,these compounds were slowly added to 40 mL of water.Following dispersion, stirring was continued for 2 min to obtain 40 mL of selatinib ditosilate suspension. After the suspension was comsumed by subjects,the container was rinsed with 40, 80, and 80 mL of water in turn and took them down.

### Sample collection and preparation

Serial venous blood samples (5 mL) of selatinib or lapatinib plasma concentrations were collected on each dosing day at the following time points: 0, 0.5, 1.0, 2.0, 3.0, 4.0, 5.0, 6.0, 8.0, 12.0, 24.0, 36.0, 48.0, 72.0, and 96.0 h after dosing.

Blood samples were collected into heparinized vacutainer tubes and gently mixed. Thereafter,plasma was separated by centrifugation (3000 rpm at 4 °C for 10 min) within 1 h of sample collection and stored frozen at −80 °C prior to analysis.

### Safety assessment

All subjects,including those in Part 1 and Part 2, were included in safety analysis. Safety was evaluated by continuous observation of adverse events (AEs; focusing on the gastrointestinal tract and hepatobiliary toxicity), vital signs, biochemistry, hematology, ECG, and urinalysis at baseline, during the trials at the established time points following drug administration (pre-dose, 1, 4, 12, 24, 48, 72, and 96 h after administration), and at follow-up visits after study completion. AEs were classified by their intensity as mild, moderate, and severe.

### Pharmacokinetic study

#### Part 1.

An early PK study^[[24^^]^ revealed that selatinib is oxidatively metabolized to the active metabolite lapatinib in vivo. Therefore, in this study, the PK of selatinib and metabolite lapatinib was simultaneously monitored in vivo. The concentrations of selatinib and lapatinib were determined by HPLC-MS/MS and expressed as the mean concentration (mean) and standard deviation (SD) at each time point. The PK parameters of selatinib and lapatinib were computed using a standard non-compartmental method on WinNonlin Professional software. Statistical analysis was used to determine the difference in the PK parameters of selatinib and metabolite lapatinib between the dose groups and gender, and whether the PK characteristics of selatinib conformed with linear kinetics.

#### Part 2.

The PK characteristics of selatinib and the metabolite, lapatinib, were monitored in subjects administered A and B, whereas those of lapatinib were monitored in subjects administered C. The concentrations of selatinib and lapatinib were determined by HPLC-MS/MS and expressed as the mean and SD at each time point. The PK parameters of selatinib and lapatinib were computed using a standard non-compartmental method with WinNonlin Professional software. The AUC ratio, C_max_ ratio, and 90% confidence interval (CI) of subjects administered A and B were calculated to compare the relative bioavailability of the A/B for formulation optimization. Exposure to the original drug and active drug in A and C was compared to determine whether selatinib can resolve the low bioavailability of lapatinib.

### Outcomes

The primary endpoints were safety, the PK profile of the single ascending doses of selatinib, and the maximum tolerated dose (MTD) in healthy subjects. Secondary endpoints were the relative bioavailability and active drug exposure to selatinib.

### Statistical analysis

Statistical description methods were used to identify the occurrence of adverse reactions.

Part 1: Analysis of Variance (ANOVA) was used to determine the difference in PK parameters between the dose groups and gender for selatinib and the metabolite lapatinib. A *P* value<0.05 was considered to indicate statistical significance. Statistical analysis of T_max_ was performed by multi-sample comparison using Kruskal-Wallis H test to determine whether there was a difference in the peak time of selatinib or the metabolite lapatinib between the dose groups. Linear regression analysis was performed on the logarithm of the AUC_0-t_, AUC_0-∞_, and C_max_ of the different dose groups of selatinib. Further, the logarithm of the dose and the regression coefficient and its 95%CI were calculated. The Kruskal-Wallis H test was performed with the dose-corrected AUC_0-t_, AUC_0-∞_, and C_max_ to investigate whether the PK characteristics of selatinib in the range of 50–500 mg were consistent with linear kinetics. Moreover, the Kruskal-Wallis H test was performed on the conversion of the metabolite, lapatinib, in each dose group to determine whether a difference existed between the dose groups.

Part 2: The AUC ratio, C_max_ ratio and 90% CI of subjects administered A and B were calculated to compare the relative availability of A/B for formulation optimization. Exposure to the original drug and active drug in A and C was compared to determine whether selatinib could resolve the clinical problem of low bioavailability of lapatinib.

## Results

### Demographic and baseline characteristics

A total of 64 subjects were enrolled in Part 1, 32 of whom were male. Of these subjects, 52 were administered the test drug, whereas 12 were administered the placebo. In Part 2, 6 subjects, all of whom were males,were enrolled. All subjects (Part 1 and Part 2) completed the study and were included in the outcome analysis. The baseline characteristics of the subjects are presented in Table 1.

**Table 1:** Demographic characteristics of test subjects

	Part 1 (*n* = 64)	Part 2 (n = 6)
Male/Female (n/n)	32/32	6/0
Age (year)	22.5 (18–29)	21.5 (18–24)
Height (cm)	164.0 ± 8.3 (150–187)	167.5 ± 5.1 (163–177)
Weight (kg)	56.8 ± 7.7 (45.0–77.0)	59.2 ± 3.9 (54.5–64.5)
BMI (kg/m^2^)	21.0 ± 1.6 (19.0–24.0)	21.1 ± 1.6 (19.1–23.7)

**Notes:** Data are the mean ± SD(min-max), except sex (male/female) is the n/n, age (year) is median (min-max)

**Abbreviations:** BMI, body mass index; SD, standard deviation

### Safety evaluations

Selatinib displayed a good safety and tolerability profile at all tested doses. As a result,the maximum tolerated dose (MTD) could not be determined. According to the randomized schedule, each subject was exposed to the test or placebo-controlled drug once. No life-threatening AEs were observed in Parts 1 and Part 2 of the study.

In Part 1, 22 AEs were observed in 19 (36.5%) of the 52 subjects administered the test drug, and 7 AEs were reported in 6 (50%) of the 12 subjects administered the placebo. Details of the AEs are summarized in Table 2. AEs were identified in the different dose groups: 50 mg (0 cases), 100 mg (3 subjects, 3 cases), 200 mg (4 subjects, 5 cases), 250 mg (3 subjects, 4 cases), 300 mg (5 subjects, 6 cases), 350 mg (2 subjects, 2 cases), and 500 mg (2 subjects, 2 cases). Of these, 14 may be related to the study drug, whereas 8 may not be related to the study drug. The AEs observed in this study were diarrhea, periodontal disease, elevated total serum bile acids, constipation, abnormal white blood cells, elevated creatine phosphokinase, and increased blood bilirubin. All AEs in the test group were of grade1–2 intensity, dose-independent, and self-resolving.

**Table 2:** adverse reaction of subjects after test drugs (part 1)

AE, n (%)	Grade	50 mg	100 mg	200 mg	250 mg	300 mg	350 mg	500 mg	ALL
		(n = 4)	(n = 10)	(n = 10)	(n = 8)	(n = 10)	(n = 6)	(n = 4)	(*n* = 52)
Diarrhea	Grade 1	0	0	0	0	1 (10.0)	0	1 (25.0)	2 (3.85)
Constipation	Grade 1	0	0	1 (10.0)	0	0	0	0	1 (1.92)
Periodontal disease	Grade 1	0	0	0	1 (12.5)	0	0	0	1 (1.92)
Hematuria	Grade 1	0	1 (10.0)	0	1 (12.5)	0	0	0	2(3.85)
Urine discoloration	Grade 1	0	0	0	0	2 (20.0)	1 (16.7)	0	3(5.77)
CPK increased	Grade 2	0	1 (10.0)	1 (10.0)	2 (25.0)	1 (10.0)	0	0	5(9.62)
Blood bilirubin increased	Grade 1	0	1 (10.0)	2 (20.0)	0	0	0	1 (25.0)	4(7.69)
Leukocytosis	Grade 1	0	0	0	0	2 (20.0)	0	0	2 (3.85)
White blood cell decreased	Grade 1	0	0	1 (10.0)	0	0	0	0	1 (1.92)
Other-TSBA increased	Grade 1	0	0	0	0	0	1 (16.7)	0	1 (1.92)

**Abbreviations:** AE, adverse event; CPK, creatine phosphokinase; TSBA, Total serum bile acids

In Part 2, 12 AEs were reported in 5 of the 6 subjects administered the study drugs. Three cases of diarrhea and 1 case of hematuria were observed in subjects administered drug A, 1 case of proteinuria and 3 cases of diarrhea were observed in subjects administered drug B, and 1 case of abdominal pain and 3 cases of diarrhea were observed in subjects administered the positive reference, drug C. Based on our findings, hematuria and proteinuria may not be relevant to the study drug. All AEs were mild.Additionally, all AEs were alleviated without treatment.

### Pharmacokinetics

**Fig. 1:**
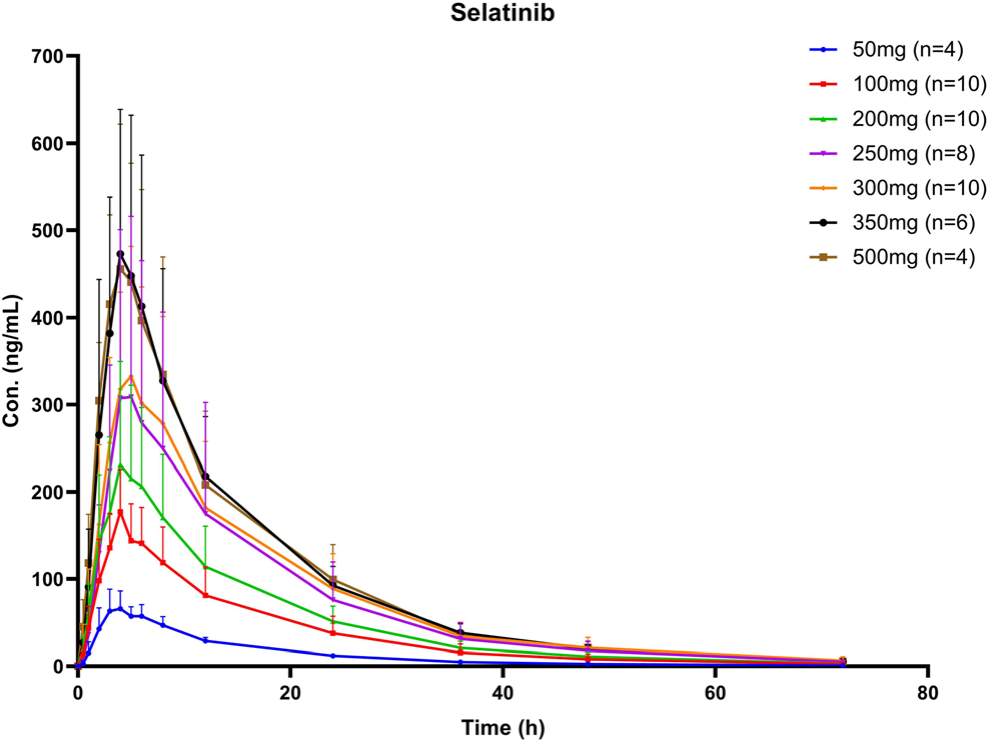
Concentration-time curves of selatinib in healthy Chinese subjects administered a single oral dose of 50–500 mg selatinib ditosilate tablet

In Part 1, the concentration-time curves of selatinib and the active metabolite, lapatinib, are presented in Fig. 1 and S2. The PK parameters of selatinib and the metabolite lapatinib in the different dosing groups are presented in Table 3 and Table S1. ANOVA revealed that the main PK parameters (C_max_, t_1/2_, AUC_0-t_, AUC_0-∞_, V/F, CL/F) of selatinib and metabolite lapatinib did not significantly differ between dose groups, except for the dose-dependent parameters (C_max_, AUC_0-t_, AUC_0-∞_). Further, there were no significant differences in the major PK parameters between genders. Kruskal-Wallis H test indicated that the differences in the T_max_ of selatinib and lapatinib among the 7 dose groups were not significant. Regression analysis of the LnC_max_, LnAUC_0-t_, LnAUC_0-∞_, and LnDose of selatinib revealed that the 95% CI of regression coefficient β was 0.572–0.993, 0.635–1.023, and 0.633–1.021,respectively (Table 4). There was no significant difference in the dose-corrected C_max_, AUC_0-t_, and AUC_0-∞_ of selatinib between the dose groups. Furthermore, the conversion rate of lapatinib, AUC_Lapatinib_/AUC_Selatinib_, did not significantly differ between the dose groups.

**Table 3:** Pharmacokinetic properties of selatinib of healthy Chinese subjects after a single 50-500 mg dose of selatinib (part 1)

PK parameter	Part 1-selatinib						
	50 mg (n = 4)	100 mg (n = 10)	200 mg (n = 10)	250 mg (n = 8)	300 mg (n = 10)	350 mg (n = 6)	500 mg (n = 4)
**C**_**max**_ **(μg/mL)**	0.0694 ± 0.0185	0.178 ± 0.0488	0.247 ± 0. 110	0.329 ± 0.211	0.352 ± 0.147	0.494 ± 0.156	0.486 ± 0.144
**AUC**_**0-t**_ **(**μg*h/mL)	0.969 ± 0.161	2.664 ± 1.039	3.708 ± 1.465	5.319 ± 3.248	5.904 ± 2.428	7.072 ± 2.001	7.142 ± 2.463
**AUC**_**0-∞**_ **(**μg*h/mL)	0.983 ± 0.165	2.688 ± 1.058	3.735 ± 1.480	5.368 ± 3.274	5.958 ± 2.461	7.115 ± 1.995	7.191 ± 2.482
**T**_**max**_ **(h)**	4.0 (3.0–6.0)	4.0 (4.0–6.0)	4.5 (4.0–8.0)	4.0 (4.0–5.0)	4.5 (4.0–6.0)	4.5 (2.0–5.0)	3.5 (2.0–5.0)
**t**_**1/2**_ **(h)**	15.8 ± 2.98	14.3 ± 2.58	14.0 ± 1.79	15.3 ± 1.67	15.1 ± 1.09	13.8 ± 2.19	14.8 ± 0.152
**λ**_**z**_ **(×10**^**−2**^ **1/h)**	4.49 ± 0.788	4.98 ± 0.917	5.01 ± 0.651	4.58 ± 0.495	4.60 ± 0.334	5.14 ± 0.765	4.68 ± 0.0482
**V/F (L)**	1193.3 ± 321.8	853.2 ± 279.8	1247.0 ± 522.4	1393.7 ± 861.8	1380.6 ± 1006.1	1059.1 ± 425.1	1650.8 ± 652.2
**CL/F (L·h**^**−1**^**)**	51.9 ± 7.99	42.1 ± 14.7	63.7 ± 30.6	62.7 ± 36.3	62.6 ± 41.4	52.5 ± 14.7	77.3 ± 30.9
**MRT**_**0-t**_ **(h)**	15.2 ± 3.1	15.9 ± 2.4	16.4 ± 1.7	17.1 ± 1.5	17.4 ± 2.3	16.0 ± 2.6	15.5 ± 0.5
**MRT**_**0-∞**_ **(h)**	16.4 ± 3.5	16.7 ± 2.8	17.1 ± 1.9	18.0 ± 1.8	18.3 ± 2.6	16.7 ± 3.0	16.2 ± 0.5

**Notes:** Values are presented as mean ± SD, except Tmax, which is the median (min-max);

**Abbreviations:** PK, pharmacokinetic; C_max_, maximum plasma concentration; T_max_, time to C_max_; AUC_0-t_, area under the concentration curve from 0 time to the last time point; AUC_0-∞_, area under the concentration curve from 0 time to infinity; t_1/2_, terminal elimination half-life; λ_z_, first-order elimination rate constant; V/F, apparent volume of distribution corrected by bioavailability; CL/F, clearance corrected by bioavailability; MRT_0-t_, mean residence time from 0 time to the last time point; MRT_0-∞_, mean residence time from 0 time to infinity; SD, standard deviation

**Table 4:** Regression analysis of selatinib’s LnCmax, LnAUClast, LnAUCinf and LnDose in the range of 50–500 mg (part 1)

		Unstandardized Coefficients		Standardized Coefficients	t	Sig.	95% CI for B	
		B	Std. Error	Beta			Lower Bound	Upper Bound
**LnC**_**max**_	(Constant)	1.354	0.560		2.420	0.019	0.230	2.478
	LnDose	0.783	0.105	0.726	7.471	0.000*	0.572	0.993
**LnAUC**_**0-t**_	(Constant)	3.847	0.516		7.452	0.000*	2.810	4.884
	LnDose	0.829	0.097	0.772	8.576	0.000*	0.635	1.023
**LnAUC**_**0-∞**_	(Constant)	3.864	0.516		7.489	0.000*	2.828	4.901
	LnDose	0.827	0.097	0.771	8.562	0.000*	0.633	1.021

**Notes:** * *P* < 0.01

**Abbreviations:** Sig., significance; CI, confidence interval; C_max_, maximum plasma concentration; AUC_0-t_, area under the concentration curve from 0 time to the last time point; AUC_0-∞_, area under the concentration curve from 0 time to infinity

### Bioequivalence assessments

After the subjects were administered A or B in Part 2, the lapatinib formed by the metabolism of selatinib was described as A-lapatinib or B-lapatinib and that of C was described as lapatinib.

**Fig. 2:**
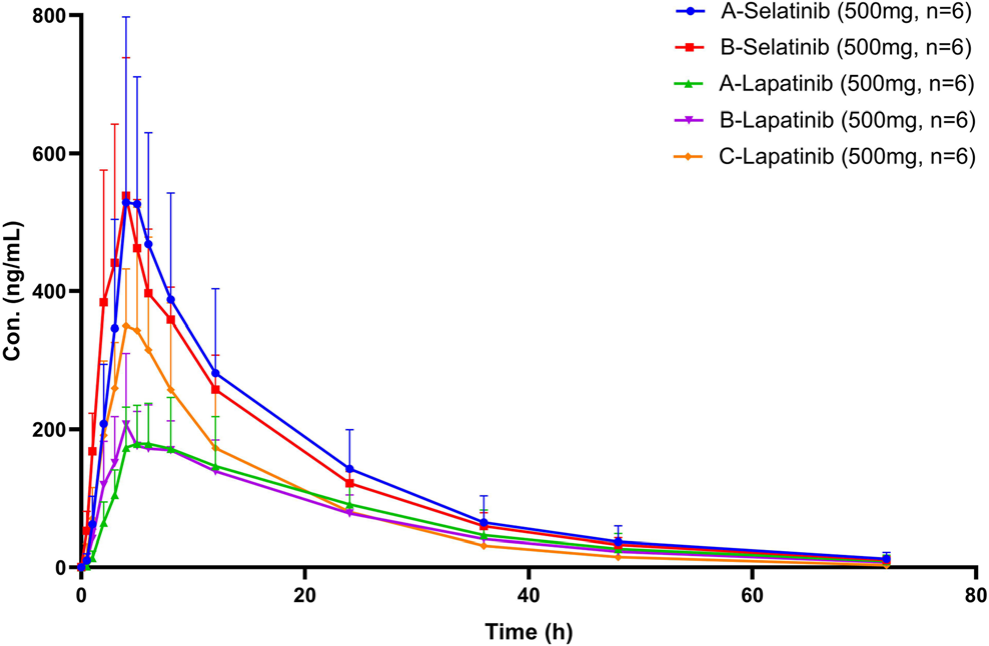
Concentration-time curves of selatinib and the active metabolite, lapatinib in healthy Chinese subjects administered a single oral 500 mg dose of A, B or C(A, selatinib ditosilate tablets; B, selatinib ditosilate dry suspension; C, lapatinib ditosylate tablets)

The concentration-time curves of selatinib and A/B-lapatinib after administration of a single dose of A or B to healthy male subjects are presented in Fig. 2 and the PK parameters are presented in Table 5. Based on statistical analysis, the PK parameters, C_max_, AUC_0-t_, AUC_0-∞_ and T_max_ of selatinib and A/B-lapatinib did not significantly differ between the different formulations. Following administration of A or B, the 90%CI of the geometric mean ratios of C_max_, AUC_0-t_, and AUC_0-∞_ of selatinib or A/B-lapatinib was not within the equivalence interval of 80–125% (Table 6).

**Table 5:** Pharmacokinetic properties of selatinib and lapatinib after a single 500 mg dose in healthy Chinese subjects taking A or B or C (part 2)

PK parameter	A (50 mg, n = 6)		B (50 mg, n = 6)		C (50 mg, n = 6)
	selatinib	A-lapatinib	selatinib	B-lapatinib	lapatinib
**C**_**max**_ **(μg/mL)**	0.601 ± 0.242	0.206 ± 0.0574	0.556 ± 0.191	0.220 ± 0.0987	0.400 ± 0.144
**AUC**_**0-t**_ (μg*h/mL)	9.28 ± 3.80	4.88 ± 2.55	8.78 ± 1.35	4.61 ± 1.56	5.52 ± 2.19
**AUC**_**0-∞**_ (μg*h/mL)	9.40 ± 3.91	4.97 ± 2.65	8.89 ± 1.41	4.68 ± 1.62	5.54 ± 2.20
**T**_**max**_ **(h)**	5.0 (4.0–6.0)	6.0 (4.0–8.0)	4.5 (3.0–6.0)	5.0 (4.0–6.0)	4.0 (2.0–5.0)
**t**_**1/2**_ **(h)**	15.6 ± 2.47	15.2 ± 3.56	16.2 ± 1.87	15.4 ± 2.11	12.7 ± 3.05
**λ**_**z**_ **(×10**^**−2**^ **/h)**	4.52 ± 0.682	4.76 ± 0.992	4.32 ± 0.453	4.58 ± 0.654	5.70 ± 1.26
**MRT**_**0-∞**_ **(h)**	19.2 ± 3.16	23.0 ± 4.86	18.9 ± 2.87	22.0 ± 3.69	15.6 ± 2.76
**V/F (L)**	1370 ± 586		1330 ± 192		1850 ± 826
**CL/F (L·h**^**−1**^**)**	62.5 ± 29.2		57.4 ± 9.04		101 ± 34.4

**Notes:** Values are presented as mean ± SD, except Tmax, which is the median (min-max);

**Abbreviations:** PK, pharmacokinetic; C_max_, maximum plasma concentration; T_max_, time to C_max_; AUC_0-t_, area under the concentration curve from 0 time to the last time point; AUC_0-∞_, area under the concentration curve from 0 time to infinity; t_1/2_, terminal elimination half-life; λz, first-order elimination rate constant; V/F, apparent volume of distribution corrected by bioavailability; CL/F, clearance corrected by bioavailability; MRT_0-t_, mean residence time from 0 time to the last time point; MRT_0-∞_, mean residence time from 0 time to infinity; A, selatinib ditosilate tablets; B, selatinib ditosilate dry suspension; A-lapatinib, the active metabolite lapatinib in subjects taking A; B-lapatinib, the active metabolite lapatinib in subjects taking B; C, lapatinib ditosylate tablets; SD, standard deviation

**Table 6:** Average bioequivalence analysis of selatinib and active metabolite lapatinib by Logarithmic transformation after a single 500 mg dose in healthy Chinese subjects taking A or B (part 2)

analyte	PK parameter	B/A%	90%CI (%)		Power (%)
			inferior limit	upper limit	
**selatinib**	**AUC**_**0-∞**_	101.09	64.07	159.48	0.1775
	**AUC**_**0-t**_	101.06	64.13	159.27	0.1779
	**C**_**max**_	94.53	55.94	159.73	0.1565
A- **Lapatinib,** B- **Lapatinib**	**AUC**_**0-∞**_	99.48	61.35	161.32	0.1679
	**AUC**_**0-t**_	99.71	61.65	161.29	0.1687
	**C**_**max**_	103.19	62.02	171.71	0.1604

**Abbreviations:** PK, pharmacokinetic; CI, confidence interval; C_max_, maximum plasma concentration; AUC_0-t_, area under the concentration curve from 0 time to the last time point; AUC_0-∞_, area under the concentration curve from 0 time to infinity; A, selatinib ditosilate tablets; B, selatinib ditosilate dry suspension; A-lapatinib, the active metabolite lapatinib in subjects taking A; B-lapatinib, the active metabolite lapatinib in subjects taking B

The concentration-time curves of selatinib, A-lapatinib, and lapatinib obtained after administration of a single dose of A or C to healthy male subjects are presented in Fig. 2. while the PK parameters are listed Table 5.

After a single oral dose of 500 mg of A was administrated to 6 healthy male subjects, the C_max_ and AUC_0-t_ of selatinib were 0.601 μg/mL and 9.28 μg*h/mL while those of A-lapatinib were 0.206 μg/mL and 4.88 μg*h/mL, respectively. After the oral administration of the same dose of C, the C_max_ and AUC_0-t_ of lapatinib were 0.400 μg/mL and 5.52 μg*h/mL, respectively. After oral administration of A (sum of selatinib and A-lapatinib), exposure of the active drug in the plasma was higher than that of the same dose of C, and the ratio after conversion based on the molecular weight of the two compounds was greater than 2 (molecular weight of selatinib, 565 Da; lapatinib, 581 Da).

## Discussion

This phase I study is the first to assess selatinib within a first-in-human study. Based on our findings, single oral selatinib doses up to 500 mg of were generally well-tolerated by healthy subjects with no severe toxicities. These findings that the efficacy of this compound can be evaluated in patients. Second, the bioavailability of selatinib tablets was similar to that of its suspensions.Third, the active drug exposure to selatinib (selatinib combined with the metabolite, lapatinib) was more than two-fold that of the same dose of lapatinib. Collectively,these findings support its further clinical development as a component in a novel anticancer drug.

Previously, the clinical utility of the ErbB inhibitor for treating breast cancer and subsets of patients with lung cancer was demonstrated [27, 28]. Lapatinib is a typical dual action TKI that exhibits activity both at the EGFR and ErbB2 receptors [29]. However,several studies have reported that lapatinib has poor aqueous solubility (7 μg/mL), limiting its clinical use [22, 23, 30]. Compared to lapatinib, selatinib has better oral absorption, higher bioavailability, and less individual variation. Furthermore, selatinib offers better security and can be clinically developed to treat gastric cancer, breast cancer, and non-small cell lung cancer, with high expression levels of EGFR and ErbB2.

The doses administered in this first-in-human study were based on multiple factors, including preclinical information, non-clinical PK and safety data, and existing knowledge of agents that target the same molecular pathways. In the initial design, dose escalation occurred from 50 to 350 mg, with 50 mg as the starting dose. Thereafter, the NOAEL was used to determine the maximun exposure dose [31]. In a long-term toxicity experiment conducted with rats, the NOAEL was 75 mg/kg. As a result, 350 mg was obtained as the climbing end point following body surface area conversion. Selection of 500 mg as the hightest dose was based on the hypothesis that 50–350 mg selatinib is safe and well-tolerated by healthy subjects in China as dose-limiting toxicity was not observed. Because a total of 33% of the threshould is commonly accepted in most phase I cancer trials [32], a tolerance test was conducted with 500 mg.

Based on our findings, selatinib was generally safe and exhibited a reasonable tolerability profile in healthy adult subjects administered a single treatment of 50, 100, 200, 350, and 500 mg. Although most subjects reported local reactions to selatinib, all reactions were of mild-to-moderate intensity and quickly resolved. According to the doctor’s judgements, statistical analysis of clinical symptoms, and the laboratory test results, the most frequent treatment-emergent AEs with selatinib was diarrhoea while the most common may be drug-related laboratory abnormality was elevated creatine kinase. Although increased creatine phosphokinase increased was observed in the test drug and placebo groups, the relationship between creatine phosphokinase increased and selatinib cannot be excluded. The AEs observed were similar to those identified in clinical trials of lapatinib [33], but QT prolongation and liver toxicity were not observed in our study [34, 35]. As liver toxicity would likely be observed only after chronic dosing and our study used only a single-dose, its liver toxicity must be further evaluated in multiple dose phases.

Following oral administration, the plasma concentration of selatinib increased with increasing doses; this increase was observed up to the hightest dose examined. Selatinib serum concentrations were found to increase in proportion with the dose. In the 50–350 mg dose group, regression analysis with LnDose as the independent variable and LnC_max_, LnAUC_0-t_, and LnAUC_0-∞_ as the dependent variables showed that the 95%CI of the regression coefficients all contained 1(Table S2), indicating linear pharmacokinetics. However, when 500 mg was added to 50–350 mg for regression analysis, it was found that the 95% CI for the regression coefficient of LnC_max_ and LnDose was 0.619–0.992, which is close to but does not include 1. This finding may be related to the small sample size of this dose group. In summary, the pharmacokinetics of selatinib administered in the range of 50–500 mg to healthy Chinese subjects conforms with linear characteristics. However, further studies are needed to confirm this finding.

The PK parameters ratios of C_max_, AUC_**0-**t_,and AUC_**0-∞**_ of selatinib and A/B-lapatinib after the administration of a single dose of A or B to healthy male subjects were not within the equivalence interval of 80% to 125% (Table 6). This finding may be related to the small number of subjects and the low degree of power. However, as the 90%CI contains “1”, the bioavailability of A and B can be considered as be similar. Unlike lapatinib, selatinib itself and its active metabolite (lapatinib) can act as anticancer compounds. Herein, we found that exposure to the active drug in the plasma after the oral administration of A (the sum of selatinib and lapatinib) was more than 2-fold higher than that of the same dose of C. One of the major issues of many anticancer drugs is their low oral bioavailability. However, selatinib is however expected to solve this issue by increasing the level of its active ingredient.

This study had some limitations. First, the PK data were only obtained from healthy Chinese subjects administered a single dose of the drug. Therefore, the PK values may differ in patients observed in clinical practice or administered other dosage regimens. Further studies of a wider population are needed. Second, the sample size employed in this phrase I study was insufficient to detect rare AEs.

## Conclusions

In summary, selatinib with high bioavailability of oral administration showed acceptable safety in a single dose and exhibited linear pharmacokinetics over the dose range studied in healthy Chinese subjects. Active exposure to selatinib was much greater than that to lapatinib, supporting its development as an adjuvant for anticancer treatment.

## Electronic supplementary material

ESM 1 (PDF 467 kb)
