# Supplementary material for: First-in-human, phase I single-ascending-dose study of the safety, pharmacokinetics, and relative bioavailability of selatinib, a dual EGFR-ErbB2 inhibitor in healthy subjects
Source: Invest New Drugs. 2020 Jun 13;38(6):1826–35. doi: 10.1007/s10637-020-00959-6 (PMC7575490; doi:10.1007/s10637-020-00959-6)
Supplement: Supplementary file 1 — (PDF 467 kb) [file 10637_2020_959_MOESM1_ESM.pdf]

Supplement

First-in-human, phase I single-ascending-dose study of the safety, pharmacokinetics, and relative bioavailability of selatinib, a dual EGFR-ErbB2 inhibitor in healthy subjects

Meng-na Wang[1*], Yun Kuang[1*], Li-ying Gong[1,2,4], Ye Hua[1] , Qi Pei[3], Cheng-xian Guo[1,2], Yu Cao[4], Jie Huang[1,2#], Guo-ping Yang[1,2,3#]

Journal name: Investigational New Drugs

Author information:

Affiliations

1 Center for Clinical Pharmacology, The Third Xiangya Hospital,Central South University, Changsha, Hunan 410013, People's Republic of China;

2 Research Center of Drug Clinical Evaluation of Central South University, Changsha, Hunan, 410013, People's Republic of China;

3 Department of Pharmacy, The Third Xiangya Hospital, Central South University, Changsha, Hunan 410013, People's Republic of China;

4 Department of Cardiology, The Third Xiangya Hospital, Central South University, Changsha, Hunan 410013, People's Republic of China

Contributions

Designed Research: Guoping Yang, Liying Gong, Jie Huang

Performed Research: Jie Huang, Mengna Wang, Yun Kuang, Chengxian Guo, Yu Cao, Ye Hua

Analyzed Data: Liying Gong, Mengna Wang, Jie Huang, Ye Hua, Qi Pei

Wrote Manuscript: Mengna Wang, Yun Kuang, Liying Gong, Guoping Yang

Language Modification: Yun Kuang, Mengna Wang

**Meng-na Wang and Yun Kuang contributed equally to this work.*

# Corresponding author

Guo-ping Yang, ygp9880@126.com; Jie Huang, cellahuang1988@163.com.

Table S1 Pharmacokinetic properties of active metabolite lapatinib of healthy Chinese subjects after a single 50-500mg dose of selatinib (part 1).

PK parameter	Part 1-active metabolite lapatinib						
	50mg (n=4)	100mg (n=10)	200mg (n=10)	250mg (n=8)	300mg (n=10)	350mg (n=6)	500mg (n=4)
**C_{max} (μg/mL)**	0.0410 ± 0.00615	0.0939 ± 0.0270	0.136 ± 0.0589	0.151 ± 0.0677	0.158 ± 0.0530	0.243 ± 0.0639	0.244 ± 0.0802
**AUC_{0-t} (μg*h/mL)**	0.715 ± 0.047	1.839 ± 0.884	2.509 ± 1.111	3.356 ± 1.820	3.420 ± 1.308	4.595 ± 1.224	4.710 ± 1.704
**$AUC_{0-\infty}$ (μg*h/mL)**	0.724 ± 0.048	1.860 ± 0.903	2.529 ± 1.120	3.390 ± 1.837	3.459 ± 1.331	4.629 ± 1.215	4.743 ± 1.713
**T_{max} (h)**	4.0 (4.0-6.0)	4.0 (4.0-8.0)	5.0 (4.0-8.0)	4.0 (4.0-6.0)	6.0 (4.0-8.0)	5.0 (3.0-6.0)	4.5 (3.0-8.0)
**$t_{1/2}$ (h)**	13.9 ± 2.41	13.6 ± 2.16	13.5 ± 1.99	14.7 ± 1.54	15.1 ± 0.782	13.7 ± 2.23	14.2 ± 0.895
**λ_z ($\times10^{-2}$ /h)**	5.08 ± 0.754	5.20 ± 0.845	5.23 ± 0.761	4.78 ± 0.522	4.61 ± 0.235	5.16 ± 0.748	4.89 ± 0.317
**V/F (L)**	1383.9 ± 199.7	1218.9 ± 419.9	1905.0 ± 1183.5	2036.8 ± 1283.0	2270.2 ± 1371.8	1644.9 ± 797.1	2398.4 ± 905.0
**CL/F ($L\cdot h^{-1}$)**	69.3 ± 4.9	64.3 ± 26.1	97.5 ± 52.3	95.1 ± 55.0	104.8 ± 62.1	80.9 ± 24.9	115.6 ± 38.2
**MRT_{0-t} (h)**	17.5 ± 3.8	18.3 ± 3.4	18.3 ± 1.8	19.8 ± 2.2	19.7 ± 2.6	18.4 ± 2.9	17.8 ± 0.9
**$MRT_{0-\infty}$ (h)**	18.6 ± 4.1	19.3 ± 3.8	19.1 ± 2.1	20.8 ± 2.4	20.8 ± 3.0	19.2 ± 3.5	18.5 ± 0.9

Notes: Values are presented as mean ± SD, except Tmax, which is the median (min-max);

Abbreviations: PK, pharmacokinetic; C_{max}, maximum plasma concentration; T_{max}, time to C_{max}; AUC_{0-t}, area under the concentration curve from 0 time to the last time point; $AUC_{0-\infty}$, area under the concentration curve from 0 time to infinity; $t_{1/2}$, terminal elimination half-life; λ_z, first-order elimination rate constant; V/F, apparent volume

of distribution corrected by bioavailability; CL/F, clearance corrected by bioavailability; MRT_{0-t}, mean residence time from 0 time to the last time point; $MRT_{0-\infty}$, mean residence time from 0 time to infinity; SD, standard deviation.

Table s2 Regression analysis of selatinib's LnCmax, LnAUClast, LnAUCinf and LnDose in the range of 50-350 mg (part 1).

		Unstandardized Coefficients		Standardized Coefficients	t	Sig.	95% CI for B	
		B	Std. Error	Beta			Lower Bound	Upper Bound
**LnC_{max}**	(Constant)	1.276	0.626		2.037	0.047	0.015	2.536
	LnDose	0.799	0.119	0.703	6.712	0.000*	0.559	1.038
**$LnAUC_{0-t}$**	(Constant)	3.640	0.567		6.422	0.000*	2.499	4.781
	LnDose	0.871	0.108	0.766	8.087	0.000*	0.654	1.088
**$LnAUC_{0-\infty}$**	(Constant)	3.657	0.567		6.454	0.000*	2.517	4.797
	LnDose	0.869	0.108	0.766	8.075	0.000*	0.653	1.086

Notes: * P<0.01

Abbreviations: Sig., significance; CI, confidence interval; C_{max}, maximum plasma concentration; AUC_{0-t}, area under the concentration curve from 0 time to the last time point; $AUC_{0-\infty}$, area under the concentration curve from 0 time to infinity.

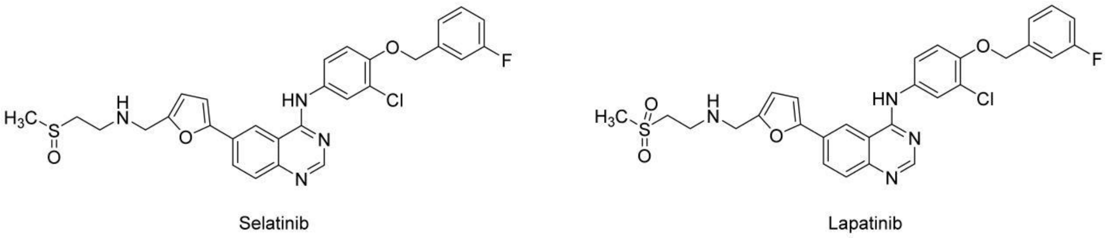

Fig. S1. Structural formula of selatinib and lapatinib

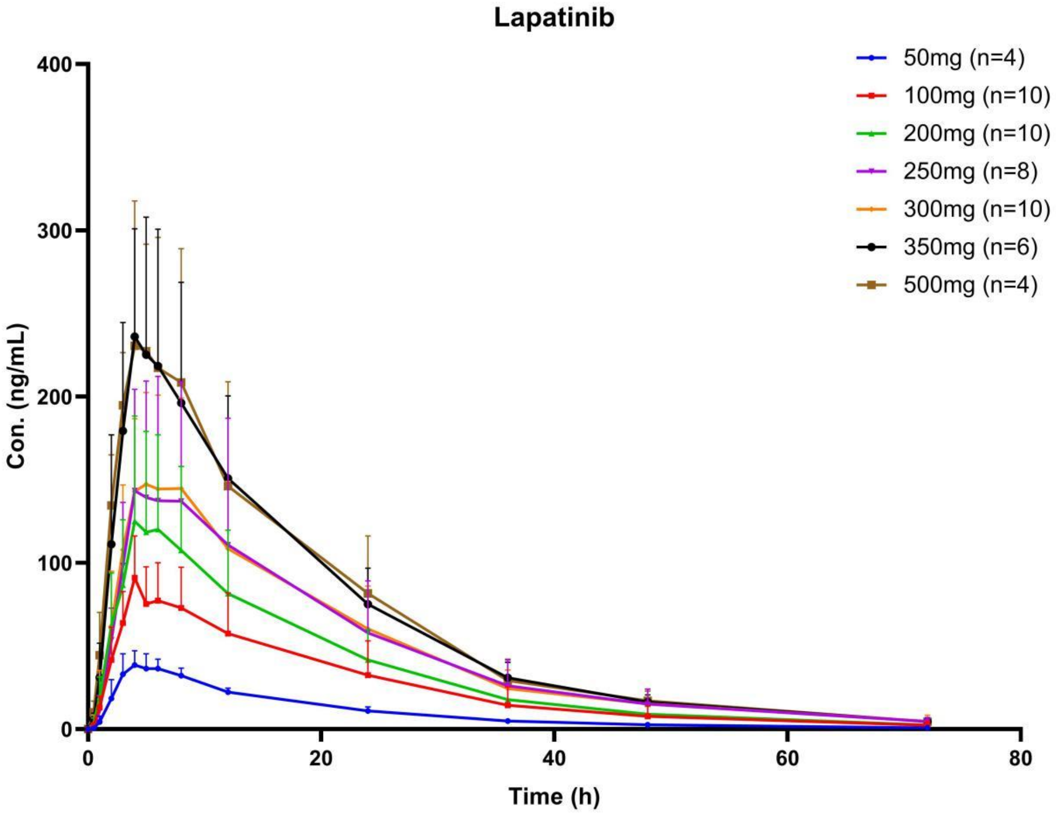

Fig. S2. Concentration-time curves of the active metabolite, lapatinib in healthy Chinese subjects administered a single oral 50–500mg dose of selatinib ditosilate tablets.

Abbreviations: Con., concentration.
